# Supplementary material for: Hidden in plain sight: A systematic review of coercion and Long-Acting Reversible Contraceptive methods (LARC)
Source: PLOS Glob Public Health. 2023 Aug 18;3(8):e0002131. doi: 10.1371/journal.pgph.0002131 (PMC10437997; doi:10.1371/journal.pgph.0002131)
Supplement: S1 File — (DOCX) [file pgph.0002131.s002.docx]

**Supplementary File 3: List of Papers**

1. Albiston, C. (1994) The Social Meaning of the Norplant Condition: Constitutional Considerations of Race, Class, and Gender. *Berkeley Women’s Law Journal.*
2. American Bar Association (1993) Norplant: Miracle Drug or Threat to Women's Rights? ”*Human Rights*
3. American College of Obstetricians and Gynecologists (2017) Committee Opinion No. 699: Adolescent Pregnancy, Contraception, and Sexual Activity.
4. American Medical Association (1992) Requirements or Incentives by Government for the Use of Long-Acting Contraceptives. *JAMA.*
5. Anonymous (2008) PCTs restricting GP access to long-acting contraception. *Pulse.*
6. Baker, L (2001) Control and the Dalkon Shield. Violence Against Women
7. Behmer Hansen RT; Arora KS (2018) Consenting to invasive contraceptives: an ethical analysis of adolescent decision-making authority for long-acting reversible contraception. *Journal of Medical Ethics.*
8. Biggs, MA; Tome, L; Mays, A; Kaller, S; Harper, CC; Freedman, L (2020) The Fine Line Between Informing and Coercing: Community Health Center Clinicians' Approaches to Counseling Young People About IUDs. *Current Opinion In Obstetrics & Gynecology.*
9. Brandao, ER; Cabral, CD (2021) Youth, gender and reproductive justice: health inequities in family planning in Brazil's Unified Health System. *Perspectives On Sexual and Reproductive Health*
10. Brandi K; Woodhams E; White KO; Mehta PK (2018) An exploration of perceived contraceptive coercion at the time of abortion. *Journal Of Women’s Health.*
11. Brandi, K; Fuentes, L (2020) The history of tiered-effectiveness contraceptive counseling and the importance of patient-centered family planning care. *Contraception.*
12. Bryson A; Koyama A; Hassan A (2021) Addressing long-acting reversible contraception access, bias, and coercion: supporting adolescent and young adult reproductive autonomy. *Culture, health & sexuality*
13. Burns, B; Grindlay, K; Dennis, A (2015) Women’s Awareness of, Interest in, and Experiences with Long-acting Reversible and Permanent Contraception*. Women's health issues.*
14. Cannon, R; White, K; Seifert, B; Woodhams, E; Brandi, K; Yinusa-Nyahkoon, L (2021) Exploring the physician's role in contraceptive counseling at the time of abortion in the US. *Contraception*.
15. Charron E, Tahsin F, Balto R, Eichelberger KY, Dickes L, Simonsen SE, Mayo RM. (2022) Provider Perspectives of Barriers to Contraceptive Access and Use among Women with Substance Use Disorders. *Women’s Health Issues.*
16. AHC (2020) Postpartum LARC: Highly Effective but Restricted by Some Hospitals. *Contraceptive Technology Update.*
17. Dasari, M; Borrero, S; Akers, AK; Sucato, GS; Dick R; Hicks A; Miller E (2016) Barriers to Long-Acting Reversible Contraceptive Uptake Among Homeless Young Women. *J Pediatr Adolesc Gynecol.*
18. De Lima Pimentel, AC; Janotti CB; Gaudenzi P; da Silva Teixeira LA. (2017) The Brief Life of Norplant® In Brazil: Controversies and Reassemblages Between Science, Society and State. *Ciencia & Saude Coletiva.*
19. Demsky, LS (1984) The Use of Depo-Provera In The Treatment Of Sex Offenders The Legal Issues. *Journal of Legal Medicine.*
20. Egan TM; Siegert, RJ; Fairley NA (1993) Use of hormonal contraceptives in an institutional setting: reasons for use, consent and safety in women with psychiatric and intellectual disabilities. *The New Zealand Medical Journal.*
21. Foster DG; Barar R;Gould H; Gomez I; Nguyen D;Biggs MA (2016)  Projections and opinions from 100 experts in long-acting reversible contraception. *Contraception*
22. Frank, ML; DiMaria C. (1997) Levonorgestrel Subdermal Implants Contraception on Trial. *Drug Safety.*
23. Gehlert, S; Lickey, S (2015) Projections and opinions from 100 experts in long-acting reversible contraception. *Contraceptive Technology Update*
24. Gilliam, ML (2015) Beyond Coercion Let Us Grapple with Bias. *Obstetrics and Gynecology.*
25. Grzanka, PR; Schuch, E (2019) Reproductive Anxiety and Conditional Agency at the Intersections of Privilege: A Focus Group Study of Emerging Adults’ Perception of Long-Acting Reversible Contraception. *Journal of Social Science.*
26. Gubrium AC; Mann ES; Borrero S; Dehlendorf C; Fields J; Arline TG; Gomez AM; Harris, LH; Higgins JA; Kimport K; Luker K; Luna Z; Mamo L; Roberts D; Romero D; Sisson G; (2016) Realizing Reproductive Health Equity Needs More Than Long-Acting Reversible Contraception (LARC). *American Journal of Public Health.,*
27. Guiahi, M (2019) Religious refusals to long-acting reversible contraceptives in Catholic settings: a call for evidence. *American Journal of Obstetrics and Gynecology.*
28. Guiahi, M; Teal SB; Swartz M; Huynh S; Schiller G; Sheeder J. (2017) What Are Women Told When Requesting Family Planning Services at Clinics Associated with Catholic Hospitals? A Mystery Caller Study. *Perspectives on Sexual and Reproductive Health.*
29. Guihai, M; Swartz M; Huynh S; Schiller G (2016) Women's Access to Family Planning Services Is Highly Variable at Obstetrics and Gynecology Clinics Affiliated With Catholic Hospitals. *Contraception.*
30. Harris LH; Wolfe, T (1995) Social and Health-Policy Concerns Raised by The Introduction Of The Contraceptive Norplant. *International Journal of Gynecology and Obstetrics.*
31. Heil S; J Higgins (2012) The Scientific and Ethical Rationale for Using Incentives to Promote Contraceptive Use Among Drug-Abusing Women. *Addiction.*
32. Hill, AL; Zachor H; Miller E; Talis J; Zelazny S; Jones JA. (2021) Trauma-Informed Personalized Scripts to Address Partner Violence and Reproductive Coercion: Follow-Up Findings from an Implementation Randomized Controlled Trial Study. *J Womens Health.*
33. Kirsch, JD; Cednoe, MA (1999) Informed consent for family planning for poor women in Chiapas, Mexico. *Lancet.*
34. Lin, YD (2011) Contextualizing Autonomy: Feminist Bioethical Perspectives on Family Planning in Taiwan. Turkiye Klinikleri Journal of Medical Sciences
35. Ma R; Cecil E; Bottle A; French R; Saxena S (2020) Impact of a pay-for-performance scheme for long-acting reversible contraceptive (LARC) advice on contraceptive uptake and abortion in British primary care: An interrupted time series study. *PLoS Medicine.*
36. Mann ES; Grzanka PR (2018) Agency-Without-Choice: The Visual Rhetorics of Long-Acting Reversible Contraception Promotion. *Symbolic Interaction.*
37. McCarthy M. (2009) ‘I have the jab so I can't be blamed for getting pregnant’: Contraception and women with learning disabilities. *Women’s Studies International Forum.*
38. McCloskey LA; Hitchcock S; Eloff I; Dinh P; Masemola K (2018) Racial and ethnic differences in patterns of long-acting reversible contraceptive use in the United States, 2011-2015. *Perspectives on Sexual and Reproductive Health.*
39. Meier S; Sundstrom B; DeMaria AL; Delay C (2019) Beyond a Legacy of Coercion: Long-Acting Reversible Contraception (LARC) and Social Justice. *Women’s Reproductive Health.*
40. Melella TJ; Travin S; Cullen K (1989) Legal and Ethical Issues in the Use of Antiandrogens in Treating Sex Offenders. *Bulletin of the American Academy of Psychiatry & the Law.*
41. Mertus J; Heller S (1992) Norplant meets the new eugenicists: the impermissibility of coerced contraception. *Saint Louis University Public Law Review.*
42. Mills, C (1999) The Ethics of Reproductive Control. *Philosophical Forum*.
43. Moniz MH; Spector-Bagdady K; Heisler M; Harris LH (2020) The role of reproductive coercion in women's risk for HIV: A case-control study of outpatients in Gauteng, South Africa. *Contraception.*
44. Morison T. (2022) Patient-provider power relations in counselling on long-acting reversible contraception: a discursive study of provider perspectives. *Culture, Health and Sexuality.*
45. Moseley CA; Beard MT. (1994) Norplant: Nursing's responsibility in procreative rights. *Nursing and Healthcare.*
46. Mubaraki, M. (1992) The Constitutionality of Court Imposed Contraception as a Condition of Probation*. Criminal Justice Journal.*
47. Nelson HL; Nelson JL. (1995) Feminism, Social Policy, And Long-Acting Contraception. *Hastings Center Report*
48. Newman, K (2019) Harm Prevention or Population Control? Amid efforts to curb the U.S. opioid crisis, a North Carolina non-profit is offering money to drug users who go on long-term birth control. *The Civic Report.*
49. Ollila, F; Hemminki E (1996) Secrecy in Drug Regulation Licensing Documentation on The Norplant® Contraceptive. *The International Journal of Risk & Safety in Medicine*.
50. Ollila, F; Hemminki E (1997) Does Licensing of Drugs in Industrialized Countries Guarantee Drug Quality And Safety For Third World Countries? The Case of Norplant Licensing In Finland. *International Journal of Health Services.*
51. Ollila, F; Koivusalo M, Hemminki E (2000) International Actors and Population Policies in India, With Special Reference to Contraceptive Policies. *International Journal of Health Services.*
52. Pam VC; Mutihir JT; Nyango DD; Shambe I; Egbodo CO; Karshima JA. (2016) Sociodemographic profiles and use-dynamics of Jadelle (levonorgestrel) implants in Jos, Nigeria. Niger Med J.
53. Parsons CD. (1990) Drugs, Science, And Ethics: Lessons from The Depo-Provera Story. *Issues in reproductive and genetic engineering*
54. Pelopida, TM; Coonrod DV; Bay RC; Campos-Outcalt D; Beaver BF; Hart E (2006) Evaluation of the Nonconsensual Placement of Intrauterine Devices in Women in Mexico. *Obstetrics and Gynecology.*
55. Persels, J. (1992) The Norplant Condition: Protecting the Unborn Or Violating Fundamental Rights? *The Journal of Legal Medicine*
56. Relias Media (2021) Study: IUD Counseling Can Appear Coercive*. Contraceptive Technology Update.*
57. Rhodes, AM (1991) Norplant and the 'coerced contraception' controversy. *MCN.*
58. Roberts, L; Kaplan (2016) Locating LARC Within the Context Of Sexual And Reproductive Justice. *American Journal of Public Health*
59. Romero L; Mendoza Z; Hurst S; Zapata LB; Powell R; Vale; Lathrop E (2020). Strategies and safeguards to ensure access to long-acting reversible contraception removal after the Zika Contraception Access Network ended: A prospective analysis of patient reported complaints. *Contraception.*
60. Lowe P, Rowlands S. Long-acting reversible contraception: Targeting those judged to be unfit for parenthood in the United States and the United Kingdom, Global Public Health
61. Rutherford, C (1992) Reproductive Freedoms and African American Women. *Yale J Law Fem.*
62. Sathyamala, C (2019) In the Name of Science: Ethical Violations In The ECHO Randomised Trial. *Glob Public Health.*
63. Satia JK; Maru RM (1986) Incentives and Disincentives in the Indian Family Welfare Program. *Studies in Family Planning.*
64. Savage, W (1982) Taking Liberties With Women: Abortion, Sterilization, And Contraception*. Int J Health Serv*
65. Scott, JA; Campos-Outcalt, D (2005) Non-consented IUD placement reported by Mexican immigrants: A caution for caregivers in the US? *Journal of Family Practice.*
66. Senderowicz L, Pearson E, Hackett K, et al. 'I haven't heard much about other methods': quality of care and person-centredness in a programme to promote the postpartum intrauterine device in Tanzania. BMJ Glob Health.
67. Senderowicz, L. Higgins, J. (2015) Reproductive Autonomy Is Nonnegotiable, even in the Time of COVID-19. *International Perspectives on Sexual and Reproductive Health*
68. Senderowicz, L. (2019) “I was obligated to accept”: A qualitative exploration of contraceptive coercion. *Social Science & Medicine.*
69. Skracic I (2020) Examining The Association Between Experiences Of Reproductive Coercion And Current Contraceptive Use. *Contraception.*
70. Smith, E; Sundstrom, B; Delay C. (2020) Listening to Women: Understanding and Challenging Systems of Power to Achieve Reproductive Justice in South Carolina. *Journal of Social Issues.*
71. Spitz, S (1993) The Norplant Debate: Birth Control or Woman Control? *Columbia Human Rights Law Review.*
72. Srinivas, KR; Kanakamala K. (1992) Introducing Norplant Politics of Coercion. *Economic and Political Weekly.*
73. Stanback, J; Twum-Baah KA (2001) Why Do Family Planning Providers Restrict Access to Services? An Examination in Ghana. *International Family Planning Perspectives.*
74. Stevenson, AJ; Flores-Vazquez, IM; Allgeyer RL; Schenkkan P; Potte JE (2016) The impact of removing Planned Parenthood from Texas Women’s Health Program. *New England Journal of Medicine.*
75. Sznajder, K; Carvajal, DN; Sufrin C. (2019) Patient perceptions of immediate postpartum long-acting reversible contraception: a qualitative study. *Contraception*.
76. Takeshita, C (2004) Contraceptive Technology And Reproductive Rights: The Iud At Historical And Geographical Junctures. [*Gendered Perspectives on Reproduction and Sexuality, Volume 8*](https://www.emerald.com/insight/publication/doi/10.1016/S1529-2126(2004)8)
77. Takeshita, C (2010) The IUD in Me: On Embodying Feminist Technoscience Studies. *Science as Culture.*
78. Takeshita, C. (2015). Biopolitics of IUD: Strategies in the Global South. *TRAVAIL GENRE ET SOCIETES*
79. Thompson, M, C (1996) Contraceptive implants: long acting and provider dependent contraception raises concerns about freedom of choice. *BMJ.*
80. Thompson, K. Kirshner HK, Irwin S, Lee A, Dineen RB, Choo S, Sufrin (2021) Perceptions of long-acting reversible contraception among women in an urban US jail. *Contraception*.
81. Tomar, S; Dehingia, N; Dey, AK; Chandurkar, D; Raj, A; Silverman, JG (2020) Associations of intimate partner violence and reproductive coercion with contraceptive use in Uttar Pradesh, India: How associations differ across contraceptive methods. *PlosOne*
82. Van Hollen, C. (1998) Moving targets: Routine IUD insertion in maternity wards in Tamil Nadu, India. *Reproductive Health Matters.*
83. Vance, JL (1994) Womb for Rent: Norplant and the Undoing of Poor Women. *Hastings Constitutional Law Quarterly.*
84. Vanthuyne A; Pittrof, F; Moscuzza F. (2015) Re: Targeted encouragement of sexually active drug and alcohol-dependent women to use long-acting reversible contraception is legitimate. *Obstetrics and Gynecologist.*
85. Volscho, TW. (2021) Racism and Disparities in Women’s Use of the Depo-Provera Injection in the Contemporary USA. *Critical Sociology.*
86. Welch, MK (2010) Not Women's Rights: Birth Control as Poverty Control in Arkansas. *Arkansas Historical Quarterly*
87. White, K; Hopkins, K; Aiken, ARA; Stevenson, A; Hubert C; Grossman D; Potter JE (2015) The Impact of Reproductive Health Legislation on Family Planning Clinic Services in Texas. *Am J Public Health.*
88. Wilson, K. (2018) For reproductive justice in an era of Gates and Modi: the violence of India’s population policies. *Feminist Review.*
89. Winters, DJ; McLaughlin, AR. (2019) Soft Sterilization: Long-Acting Reversible Contraceptives in the Carceral State. *Affilia: Journal of Women & Social Work.*
90. Woo, JC;. Alamgir, H; Potter JE. (2016) Women’s experiences after Planned Parenthood’s exclusion from a family planning program in Texas. *Contraception.*
91. Young M (1995) Reproductive Technologies and the Law: Norplant and the Bad Mother. *Marriage & Family Review.*
92. Zeal C; Higgins JA; Newton SR (2018) Patient-Perceived Autonomy and Long-Acting Reversible Contraceptive Use: A Qualitative Assessment in a Midwestern, University Community. *Biores Open Access.*
