# Supplementary material for: Hidden in plain sight: A systematic review of coercion and Long-Acting Reversible Contraceptive methods (LARC)
Source: PLOS Glob Public Health. 2023 Aug 18;3(8):e0002131. doi: 10.1371/journal.pgph.0002131 (PMC10437997; doi:10.1371/journal.pgph.0002131)
Supplement: Attachment — Submitted filename: Boydell PLOSGPH Response to Reviewers.docx [file pgph.0002131.s003.docx]

**Response to Reviewers**

**Journal Comments:**

1. **Please review your reference list to ensure that it is complete and correct. If you have cited papers that have been retracted, please include the rationale for doing so in the manuscript text, or remove these references and replace them with relevant current references. Any changes to the reference list should be mentioned in the rebuttal letter that accompanies your revised manuscript. If you need to cite a retracted article, indicate the article’s retracted status in the References list and also include a citation and full reference for the retraction notice.**

*We have checked all the references in the list to ensure that all the papers are available and have not been retracted.*

**2. Please send a completed 'Competing Interests' statement, including any COIs declared by your co-authors. If you have no competing interests to declare, please state "The authors have declared that no competing interests exist". Otherwise please declare all competing interests beginning with the statement "I have read the journal's policy and the authors of this manuscript have the following competing interests:"**

*This has now been added.*

**3. Please amend your detailed Financial Disclosure statement. This is published with the article. It must therefore be completed in full sentences and contain the exact wording you wish to be published.**

**a. State the initials, alongside each funding source, of each author to receive each grant.
b. State what role the funders took in the study. If the funders had no role in your study, please state: “The funders had no role in study design, data collection and analysis, decision to publish, or preparation of the manuscript.”
c. If any authors received a salary from any of your funders, please state which authors and which funders.**

**If you did not receive any funding for this study, please simply state: “The authors received no specific funding for this work.”**

*This has now been added.*

**4. Please provide separate figure files in .tif or .eps format only and remove any figures embedded in your manuscript file. Please also ensure that all files are under our size limit of 10MB.**

For more information about figure files please see our guidelines:

[https://journals.plos.org/globalpublichealth/s/figures](https://linkprotect.cudasvc.com/url?a=https%3a%2f%2fjournals.plos.org%2fglobalpublichealth%2fs%2ffigures%26nbsp%3b&c=E,1,dtqeaEfSm1yYcOChf5rHvAWo7iTf2__0RwImSg8qpxRjt8Uz77i0f2b6Q8_LSroXug_NwS0_eOmlRDF6GZf7DiHr1WryPxKOhseuCja6ST7_GuRjQA,,&typo=1&ancr_add=1)
[https://journals.plos.org/globalpublichealth/s/figures#loc-file-requirement](https://linkprotect.cudasvc.com/url?a=https%3a%2f%2fjournals.plos.org%2fglobalpublichealth%2fs%2ffigures%23loc-file-requirement&c=E,1,ARizUerDhrNFfbyfivUXXBkAjjXGz0azyYSwOkq43ll5WCLEHCiMt4jcx05_C1EhY0CCCFoa7CS4KIb5PUQ2WdTJOQdjj8oLvVDBQxCkqNZ6f_IP&typo=1&ancr_add=1)

**5. We have noticed that you have uploaded Supporting Information files, but you have not included a list of legends. Please add a full list of legends for your Supporting Information files after the references list.**

The figures have been removed from the main document and saved as TIFF files

Reviewers' comments:
**Have the authors made all data underlying the findings in their manuscript fully available (please refer to the Data Availability Statement at the start of the manuscript PDF file)?**

One reviewer commented that we have not made this data available, but we have provided a detailed table of the papers that were included in the analysis. See File 3.

**Reviewer #1: I believe that the research is sound and the publication is ready. The only hesitation I have is that the data is drawn from publications dating back to the 1980s and 1990s, which may not be directly comparable with writings from the 2000s because the discourse of reproductive choice has changed quite a bit. In some ways the contexts have been lost.**

This is a fair point, and our analysis has not been presented with sufficient nuance around the comparability of data. We have pointed to this in the limitations and revised the text to better reflect the particularities of the period that a subject was covered. Specifically, we have made note of the temporal aspects of each metanarrative in the “Characteristics of metanarratives” in Table 3 and in each metanarrative section in “overview of metanarratives.” In doing this, we aim to add a general point to the characteristic of each metanarrative to show the underlying time frame that helped to give shape to that metanarrative. Further, we have made note of this in the additional paragraph under the ‘continuum of coercive force’ section to argue that while there are temporal trends to the categories we have created in the continuum of coercive force, the time when these coercive practices took place is also not as strictly bound to the different categories of the continuum as it is in the metanarrative categories.

**Reviewer #1: Another point I would like to bring up is that "coerced contraception" and the "coerced non-access to contraception" appears in the same section even though these have opposite repercussions. I felt that they should be separated for clarity.**

Thank you, we can see the need to clarify the difference between ‘coerced contraception’ and ‘coerced non-access to contraception.’ We have clarified that we collected both types of coercion in the methodology and added the sentence “We also included papers in which coercion was used to force women to use LARCs as well as papers in which women were forcibly denied access to LARCs.” Further, in the results section, in the sub-section “Laws and policies,” we have added a similar sentence that explicitly draws out this difference as we transition from describing coerced contraception to coerced non-access to contraception.

**Reviewer #2: The authors have presented a review meta-narrative with title "Hidden in plain sight: A systematic review of coercion and Long-Acting Reversible Contraceptive Methods (LARC)". The study concept is relevant to public health and future policy and programme on contraception as regards LARCs. The methods adopted for the review are standard and novel while the general write-up is clear and addressed the aim of the review. In general, the review is limited by the available evidence which is skewed to the US and Europe leaving a large lacuna with the LMICs. The introduction section should have background review of the dimension (Law, public health and social) to LARC coercion.**

Thank you for this comment, we are not completely clear on the reviewer’s comment here. However, we have understood this comment to mean that we need to explain why we have focused on searching the disciplines of law, public health and social science. We have added the following sentences to clarify why we have chosen these disciplines. In the introduction we stated: *“Across the legal, public health and social science literature we have found accounts….*” and have also a sentence in the methodology section stating “*We conducted a meta-narrative review (MNR) of papers documenting human rights violations involving LARCs across three disciplines where we had found existing accounts of violations.*” We have also added a sentence to the limitations section to recognise the bias this may introduce: *“The review was limited to three broad disciplines this may have affected what papers were found and included in this analysis.”* To the point that there were few papers accounting for coercion in LMICs, we explicitly looked globally for relevant papers and did not find many and, as outlined in the limitations, we fully recognise this is the result of epistemic injustice(s) in the limitations section.

**In addition, The authors needs to provide clear evidence on when contraceptive service provision becomes coercive towards LARC as against when it is based on empirical decisions. The narratives in the result are generally skewed towards coercion irrespective of underline empirical choice of LARCs in the instances given. The authors need to clearly draw a line when the choice of LARCs becomes empirical option and when it is unduly coercive. This is important for a balanced narration.**

Thank you, this is an important point and we initially tried to be clear that these were instances of coercion not when there was clinical indication for the use of LARCs. We made edits to clarify this better. In the introduction, “For others, LARCS may be the only clinically appropriate contraceptive method due to pre-existing medical conditions.” We also added a clarification in the methods section “Papers that relate to women being denied or encouraged to use LARCs on clinical grounds.”

**The authors discussed mostly the review process rather than discussing the findings of the meta-narratives. For instance, the readers will expect to see implications for different spectrum of the coercion and how they apply to future contraception programming.**

The reviewer provides some useful insights on the discussion section. To best respond to this, we have edited the discussion that focuses on review and added some text on the relevance of the findings for future programming. Though we do not have the evidence to suggest what could be applied in future contraceptive programming, we have added some recommendations for thinking about how to address this in future programming. Please see the end of the discussion section: *“In some cases, LARCs have become a standard feature of international and national SRHR programmes and by design, these programmes intentionally target marginalised communities and therefore, any failures inevitably impact these communities, this may not be an act of discrimination. However, the relative lack of power and capacity of women to give their full and informed consent and defend their rights as they receive those programmes, requires that active steps are put in place - additional and specific precautions - to explicitly ensure that the programme is NOT an instrument of discrimination, not reinforcing disadvantage and discrimination. A path to achieve this is to uplift the agency of the population(s) being served i.e. by respecting and upholding their rights to active, free, prior and informed decision-making as they - without fear or favour - choose LARCs from among other methods options or none at all. First and foremost, this requires recognizing and responding to the potential for coercion in LARC programming and ensuring appropriate safeguards are put in place to protect against such abuses in key guidance documents. This may require further research on what are effective ways to actively identify and address coercion in contraceptive programming. This may require collecting such information in Client Exit Interviews as part of quality assurance, including additional provisions in guidance on contraceptive counselling, and/or developing safe and effective reporting mechanisms. Another promising application is patient-centred care, which is defined as care that is respectful of and responsive to an individual’s preferences, needs, and values over programmatic or biomedical priorities (Dehlendorf 2013; Dehelndorf 2016). Patient-centred care focuses on high-quality and meaningful patient-centred interpersonal communication through which patients express their needs and preferences and providers actively facilitate their preferences. This requires training and support of health care professionals in patient-centred care and shared decision-making models, and incentivizing patient-centred counselling in supervision, facility, and programme assessment.”*
