# Supplementary material for: Hidden in plain sight: A systematic review of coercion and Long-Acting Reversible Contraceptive methods (LARC)
Source: PLOS Glob Public Health. 2023 Aug 18;3(8):e0002131. doi: 10.1371/journal.pgph.0002131 (PMC10437997; doi:10.1371/journal.pgph.0002131)
Supplement: Attachment — Submitted filename: Response to reviewers PLOSGPH MAy.docx [file pgph.0002131.s004.docx]

**Response to reviewers comments**

**Please review your reference list to ensure that it is complete and correct. If you have cited papers that have been retracted, please include the rationale for doing so in the manuscript text, or remove these references and replace them with relevant current references. Any changes to the reference list should be mentioned in the rebuttal letter that accompanies your revised manuscript. If you need to cite a retracted article, indicate the article’s retracted status in the References list and also include a citation and full reference for the retraction notice.**

Victoria Boydell and Robert Smith have both separately checked the reference list to ensure it is correct and complete.

**Reviewer #3: All comments have been addressed**

Thank you.

**Reviewer #4: I congratulate the authors for a very important work done in a very intelligible and articulated manner. There are not additional comments but one which I am sure it was just a typo. On Page 13 there is a place where there was supposed to be a figure as t is mentioned 'insert figure 2'. I would recommend to double check for any such mistakes and proof read the manuscript before the final acceptance for publication.**

This is not a typo, but where we intend for the figure we submitted to be inserted when the paper is production. Both Victoria Boydell and Robert Smith have both separately proofread the paper to find any remaining typographical errors.

**Reviewer #5: "A systematic review of coercion and Long-Acting Reversible Contraceptive Methods (LARC)" is important for many reasons. This review is useful for academic and practical purposes.

LARC coercion is sensitive reproductive health issue. Uninformed reproductive decisions are coercion. Understanding LARC coercion's prevalence and impact is essential for reproductive autonomy and free contraceptive choice. This systematic review can illuminate the situation, identify risk factors, and suggest policies to eliminate coercion and protect reproductive rights.**
 **Systematic reviews increase data quality. This systematic review analyses and synthesises coercion and LARC evidence. Methodological rigour eliminates bias and provides a thorough view of the issue. Thus, governments, clinicians, and researchers can trust the findings for informed decision-making, clinical practise, and further research.

Third, this extensive review enhances field scholarship. Reproductive health coercion demands constant research and analysis. Sharing this review's findings may encourage discussion, research collaborations, and a better understanding of coercion's sources and effects. This publication can encourage research on evidence-based therapies, policies, and guidelines to reduce coercion and enhance reproductive autonomy.**

Thank you for your feedback on the topic and we agree it is a critically important topic.
